# Therapeutic Potential of Human Fetal Mesenchymal Stem Cells in Musculoskeletal Disorders: A Narrative Review

**DOI:** 10.3390/ijms23031439

**Published:** 2022-01-27

**Authors:** Insun Song, Jongseop Rim, Jaemin Lee, Inseok Jang, Bosung Jung, Kisoo Kim, Soonchul Lee

**Affiliations:** 1 Department of Orthopedic Surgery, CHA Bundang Medical Center, School of Medicine, CHA University, Pocheon 13496, Gyeonggi-do, Korea; song1009@gmail.com (I.S.); jaemin0011@gmail.com (J.L.); isjamg21@naver.com (I.J.); greentea1bo@gmail.com (B.J.); kevinkisoo@hotmail.com (K.K.); 2 Fetal Stem Cell Research Center, CHA Advanced Research Institute, Seongnam 13488, Gyeonggi-do, Korea; rimjs@chamc.co.kr

**Keywords:** mesenchymal stem cells, fetal, musculoskeletal disorders, regeneration

## Abstract

Mesenchymal stem cells (MSCs) have emerged as a promising therapeutic approach for diverse diseases and injuries. The biological and clinical advantages of human fetal MSCs (hfMSCs) have recently been reported. In terms of promising therapeutic approaches for diverse diseases and injuries, hfMSCs have gained prominence as healing tools for clinical therapies. Therefore, this review assesses not the only biological advantages of hfMSCs for healing human diseases and regeneration, but also the research evidence for the engraftment and immunomodulation of hfMSCs based on their sources and biological components. Of particular clinical relevance, the present review also suggests the potential therapeutic feasibilities of hfMSCs for musculoskeletal disorders, including osteoporosis, osteoarthritis, and osteogenesis imperfecta.

## 1. Introduction

Mesenchymal stem cells (MSCs) have promising therapeutic applications as they are known to promote tissue regeneration [1]. Over 40 years ago, MSCs were successfully obtained from bone marrow [2] and various tissues and organs, such as bone [3], umbilical cord [4], adipose tissue [5], skeletal muscle [6], synovium [7], and amniotic fluid [8]. Expanded MSCs differentiate into different cell types such as osteoblasts, chondrocytes, adipocytes, myocytes, epithelial cells, endothelial cells, and neurons, all of which are applied to treat multiple diseases. Although it is difficult to compare the results of many studies, each using different isolation methods and cell-culture environments, many studies generally follow a similar standard procedure, which has led to promising results and ongoing clinical trials in the field of MSCs.

To combat this inconsistency in experimental procedures, the International Society for Cell and Gene Therapy, established in 1992 for the translation of cell and gene therapy research to a clinical setting, proposed a minimal criterion for defining MSCs [9]. According to these criteria, MSCs must express CD105/endoglin, CD73/ecto 5’-nucleotidase, and CD90/Thy-1. Furthermore, they should lack the expression of CD45/LCA, CD34, CD14 or CD11b, CD19 or CD79α, and the major histocompatibility complex (MHC) class II cell surface receptor human leukocyte antigen DR isotype (HLA-DR) [9]. The low expression of MHC class II and costimulatory molecules in MSCs is responsible for their immune privileged status [1]. Because the patient’s immune response and immune evasion strategies are critical for clinical applications, MSCs should maintain immune privilege with safe healing and clinical regeneration properties.

Musculoskeletal disorders refer to any damage or injury to the bone, muscle, cartilage, tendons, ligaments, and nerves that affect human activity or the musculoskeletal system. These disorders include osteogenesis imperfecta, nonunion, cartilage damage, osteoporosis, muscle/tendon strain, rotator cuff tendonitis, ligament sprain, and digital neuritis. Clinical attention using MSCs has gradually earned its significance as an essential therapeutic approach for treating and healing musculoskeletal and related rare diseases. Among the various types of MSCs, human fetal MSCs (hfMSCs) are far superior and show several advantages over adult MSCs. These include exceptional immunosuppressive [10], anti-inflammatory [11,12], and proliferative effects [13,14], along with greater colony-forming and osteogenic differentiation capacity [15], osteogenic gene expression [16], and longer telomere length [17]. In fact, upon exposure to interferon-gamma, hfMSCs show lower expression of HLA class I and II than that of adult MSCs [11,12]. Additionally, without apparent change in phenotype, population doublings of hfMSCs were approximately double those of adult MSCs [13,14]. 

Because hfMSCs can be isolated from various tissues and organs, such as bone marrow, liver, lung, kidney, skeletal muscle, pancreas, dermis, thymus, placenta, amniotic fluid [10], and calvaria, they may apply in various fields and diseases. If there are similarities between the origin of MSCs and the target tissue, such MSCs will likely be suitable for the target disease. From this point of view, we believe hfMSCs are one of the best healing sources available. Further studies on hfMSCs are required, as much as other types of MSCs, to understand their different aspects like homing without cell loss, immunomodulatory function with paracrine effects, differentiation and proliferation, and the signaling pathway of their unique healing mechanism. These will provide us with better opportunities to overcome various diseases. Although fetal MSCs have more advantages than adult MSCs, very few studies have reviewed the characteristics of hfMSCs compared to adult MSCs. Here, we describe the therapeutic function of fetal MSCs by reviewing their sources, biological features, and clinical trial results; by discussing the future scope of hfMSCs for the treatment of musculoskeletal disorders.

## 2. Biological Feature of Human Fetal Mesenchymal Stem Cells (hfMSCs)

### 2.1. Sources of hfMSCs 

As mentioned above, hfMSCs have been isolated from various tissues and organs, including fetal blood [13,14], bone marrow, liver [13,18], lung [18,19], pancreas [20,21], dermis [22], thymus [23], placenta and amniotic fluid [8,10,24,25,26], and calvaria. All these hfMSCs from different sources showed common MSC marker expression but had unique phenotypes. MSCs differentiate into osteogenic, chondrogenic, myogenic, adipogenic lineages, and neural cells [10,27]. However, their properties differ depending on their origin. Initially, hfMSCs were isolated from first-trimester fetal blood, liver, and bone marrow, and they had similar growth patterns and immunophenotypes [13]. Meanwhile, hfMSCs derived from the bone marrow, liver, and lungs had higher adipogenic potential than hfMSCs derived from the fetal spleen [18]. Similarly, the osteogenic differentiation properties of fetal bone marrow MSCs are greater than those of fetal liver cells [18]. hfMSCs derived from the second (2nd)-trimester fetal lungs can differentiate into osteogenic and adipogenic lineages [18]. Human fetal lung-like MSCs have maintained over 40 passages without changes in their proliferation ability, morphology, and expression of cell markers like CD13, CD29, CD44, and general MSC markers like CD90 and CD105 [19]. 2nd-trimester pancreatic MSCs appeared positive for CD44, CD29, CD13, and type I collagen but negative for CD34 and HLA class II [20]. Pancreas-derived MSCs proliferated for up to 30 passages and were also differentiated into osteocytes, chondrocytes, and adipocytes through adequate induction [20]. Zhang et al. [21] cultured pancreatic islet endocrine cells—negative for CD34, CD45, and HLA-DR—which were isolated from the fetal pancreas. Rapidly expanded MSCs derived from human fetal dermis differentiated into bone, fat, and nerve, and expanded up to 70 population doublings [22]. Second-trimester hfMSCs from the thymus were cultured to form colonies and differentiated into chondrogenic, osteogenic, myogenic, and adipogenic lines, under different conditions of each induction [23]. The phenotype and multilineage potential of hfMSCs derived from second-trimester amniotic fluid were comparable to those of adult MSCs [24,25]. Furthermore, hfMSCs derived from amniotic fluid have shown the potential to differentiate into cells with neuronal-like morphologies [26]. Amniotic membrane-derived MSCs exhibit cardiomyocyte characteristics and express the cardiac-specific transcription factor GATA4 [28]. Recently, hfMSCs derived from fetal calvaria have been discovered and characterized. These cells expanded rapidly in vitro for up to 24 passages without showing any changes in proliferation ability, morphology, and expression of specific MSC markers: positive expressions of CD105, CD90, CD44, CD29, along with negative expressions of embryonic stem cell markers SSEA-3, TRA-1-81, and hematopoietic stem cell (HSC) markers CD34 and CD45 (author’s unpublished data) (Table 1).

### 2.2. Biological Components of hfMSCs

While all hfMSCs have similar physical features, they are characterized by differential morphological phenotypes and genotypes, immune function, proliferation, differentiation capacity, and aging, depending on their origin. During MSC expansion, they show fibroblastic morphology, form colonies, and express stem cell indicators in the culture system. Like adult MSCs, hfMSCs also have molecular genotype and CD expression, even though their expression shows different characteristics depending on their origin. While expression of CD45, CD14, CD68, CD34, CD38, CD31, and HLA-DR is lacking in hfMSCs, expression of CD29, CD44, CD54, CD106, CD105, CD73, CD13, CD90, CD49b, vimentin, laminin, and fibronectin is generally observed. Interestingly, type I collagen is expressed in hfMSCs derived from the pancreas and amniotic fluid but not in those derived from bone marrow and liver [10]. Calvaria-derived hfMSCs showed positive expressions of CD105, CD90, CD44, and CD29 and negative expressions of CD45 and CD34 (author’s unpublished data). Furthermore, immune reactive molecules such as HLA Class I and HLA-DR are either not expressed or were very low in their expression in hfMSCs than in adult MSCs. This important characteristic of hfMSCs sets them apart from other MSCs. The hfMSCs derived from blood, liver, and bone marrow express adhesion molecules, including CD29, CD44, CD106/VCAM-1, CD105/endoglin, and CD73. The undifferentiated state of these MSCs is uniformly positive for intracellular markers such as fibronectin, laminin, vimentin, and mesenchymal markers such as SH2, SH3, and SH4 [13]. However, they lack the expression of CD45, CD34, CD14, CD68, and CD31 [13]. While MSCs account for a small proportion of the stem cell population, they are more prevalent during the fetal stage than adulthood. MSCs account for 1: 3000 blood cells and 1:400 bone marrow cells during the second trimester [8,13]. As a result, fetal MSCs are more abundant in tissues than adult MSCs.

### 2.3. Immunomodulation of hfMSCs

One of the essential features of MSCs is their hypoimmunogenic property that helps avoid allogeneic rejection [29]. The HLA system is a cell-surface protein responsible for regulating the immune system [30]. The expression of HLA class I by MSCs is vital for protecting them from specific NK cell deletion mechanisms [29]. However, conflicting evidence exists about the expression of MHC class I by MSCs. While Yokoyama [31] has reported that fetal MSCs do not express classical HLA class I molecules, Anker et al. [18] have reported that MSCs derived from all tested fetal tissues express HLA class I [18]. Furthermore, fetal liver MSCs have been reported to express HLA class I, but not HLA class II. However, HLA class II (HLA-DR) expression could be induced in hfMSCs after 7 days of interferon-gamma (IFN-γ) exposure compared to after 1 d in adult MSCs [11,12]. Neither undifferentiated nor differentiated MSCs elicited an immunological response. Even after stimulation with IFN-γ, hfMSCs did not activate lymphocytes [11]. These observations suggest that hfMSCs do not escape alloreactivity and suppress lymphocytes because they lack HLA class II antigens. Therefore, the exact mechanism by which MSCs exert their immunosuppressive effects remains unclear [10]. Initially, hfMSCs appeared less immunogenic than adult MSCs [32]. MSCs secrete active molecules that contribute to biologically beneficial effects on damaged tissues and organs [33] by enhancing tissue regeneration and regulating fibrosis, apoptosis, and inflammation [12,34]. Additionally, MSCs can directly inhibit the proliferation of natural killer and cytotoxic T cells [35]. Specifically, MSCs increase regulatory T cells and indirectly decrease the activity of cytotoxic T cells [36]. Thus, MSCs have a dual function in immunomodulation: immunosuppression and the regulation of inflammatory factors such as immune cells with paracrine effects. However, the paracrine effects of hfMSCs remain to be investigated. The immunological characteristics of hfMSCs are summarized in Table 2.

### 2.4. Summary

Investigation of the various origins of hfMSCs and their biological properties provide the therapeutic application of the appropriate clinical treatment of various human diseases. Although the immune reaction of hfMSCs remains unclear [10], secretion molecules of hfMSCs, including immunosuppressive factors, contribute to tissue regeneration and regulate apoptosis and inflammation [12,32,33,34], suggesting lower side effects in case of clinical applications.

## 3. Preclinical and Clinical Investigation of hfMSCs

### 3.1. Feasibility of hfMSCs for the Treatment of Musculoskeletal Disorders

Proliferation, differentiation, environmental conditions, and aging of MSCs influence their function. Fetal blood-derived MSCs are readily expandable in vitro with a population doubling time of 24–30 h, compared to at best 48–72 h for their adult counterparts, and display no apparent change in phenotype after 20 passages or 50 population doublings [13,14]. The hfMSCs not only have approximately twice the colony-forming unit-fibroblast capacity of adult MSCs, which is an essential MSC characteristic, but this also translates into superior osteogenic capacity, with higher levels of calcium deposition and alkaline phosphatase activity [15]. Guillot et al. [16] compared the basal expression of osteogenic genes in the first-trimester liver, blood, and bone marrow MSCs to adult bone marrow MSCs and found that hfMSCs had higher expression levels of all 16 osteogenic genes. Under appropriate conditions, they produce a broad spectrum of differentiated connective tissues, including bone, cartilage, adipose tissue, and myelosupportive stroma. 

However, it has been suggested that their differentiation capacity varies depending on the tissue source [18,37]. The hfMSCs can also differentiate into skeletal muscle [38,39,40] and adipocytes [41]. Choi et al. [42] showed an intriguing application of hfMSCs wherein they used fetal cartilage-derived cells for cartilage regeneration. They suggested that fetal cartilage-derived progenitor cells have stem cell properties to some extent and are more active in terms of proliferation and chondrogenic differentiation than young chondrocytes or other MSCs [42]. Adult tissue-derived MSCs lose multipotency after 20–40 passages in culture and either commit to the osteoblast lineage or undergo senescence [43,44]. This MSC aging process is more observable with increased culture time and donor age [43,45,46]. Older HSCs have a diminished self-renewal capacity and decreased numbers of progeny cells. Similar qualitative effects of aging have been observed with MSCs [45]. Further evidence for the aging of MSCs comes from studies of osteoblasts. It was observed that cell proliferation, levels of osteoblast markers, telomere length, and replicative lifespan all decreased with increasing bone age [17]. In addition, telomere length was longer in MSCs derived from fetal tissues than adult tissues. These findings imply that hfMSCs should have an advantage over adult stem cells in cell replacement therapies [10]. 

As mentioned above, similar to adult MSCs, hfMSCs are isolated from various tissues. However, compared to adult MSCs, they are found at a higher frequency in the different tissues [13,18]: especially in the case of the MSC populations derived from the amniotic fluid, placenta, and umbilical cord [47,48]. Altogether, fetal MSCs have an advantage over adult MSCs in proliferation (passage, population doubling time, senescence), differentiation (osteogenic, chondrogenic, and adipogenic lineages), stemness (colony-forming), and immune response (immune evasion, paracrine effect). Therefore, priority should be given to hfMSCs in clinical trials. 

The musculoskeletal disorders, including osteoporosis, osteoarthritis, and osteogenesis imperfecta, are expected to be improved by the treatment of hfMSCs. First, the excellent differentiation and proliferation ability of hfMSCs should be helpful for bone regeneration. Second, inflammation in osteoarthritis is a major factor associated with cartilage loss and symptoms of the disease such as joint pain, swelling, and stiffness, indicators of synovitis [49]. In this case, hfMSCs would control inflammation through the paracrine effect of hfMSCs anti-inflammatory factors, even though the study of secretion molecules of hfMSCs requires further investigation. Third, osteogenesis imperfecta, commonly diagnosed prenatally, is a disorder of type 1 collagen with a prevalence of 1/20,000 [32]. To date, two case studies of prenatal transplantation of allogenic human first-trimester liver-derived MSCs in type III and type IV osteogenesis imperfecta patients have been published [50,51]. Although there have been very few clinical trials, the products show no new fracture and improved growth velocity [50,51]. These results support the future possibility of hfMSCs for developing regenerative therapeutics.

### 3.2. Preclinical Research of hfMSCs

One of the most critical characteristics of MSCs is their ability to secrete molecules for cell-to-cell communication. They interact with target cells with the help of this secretome, which includes molecules for inflammatory response, growth factors, and the senescence-associated secretory phenotype. 

Wang et al. analyzed the fetal MSC secretome and suggested that the autocrine/paracrine effect of hfMSCs may have contributed to their enhanced proliferation and differentiation abilities [52]. They also found that the fetal MSC secretome treatment significantly reduced senescence-associated β-galactosidase expression and activity, and enhanced cell proliferation and osteogenic differentiation potential of adult MSC secretome [52]. Xu et al. described the immunogenicity of using the human MSC secretome on rat cells and the effects of the secretome on osteogenic differentiation of rat bone marrow-derived MSCs [53]. Arjmand et al. attempted co-transplantation of hfMSCs and HSCs in type 1 diabetic mouse model. They suggested that hfMSCs are a valuable source for cell therapy and that co-transplantation of MSCs can improve the therapeutic effects of HSCs [54]. Based on these preclinical investigations showing that hfMSCs improve replicative senescence and can differentiate into cardiac lineages [55], the role of fetal MSC secretome should further be investigated in clinical trials: especially for distraction osteogenesis and in type 1 diabetic mice [52,53,54]. Exosome-based research and therapies are expected to promote MSC functions such as proliferation, differentiation, and immune reactions. MSC-exosomes have been recognized as powerful tools in bionanomedicine, wherein they are involved as nanocarriers, for drug loading, and for tissue engineering [56]. MSC-exosomes have broad applications due to their regenerative and immunomodulatory properties [57,58,59,60,61]. hfMSCs-exosome also has these similar properties by canonical secretory proteins such as cytokines and growth factors. In recent years, hfMSC-exosomes have been reported in various fields such as angiogenesis [62], cutaneous wound healing [63], and impaired natural killer cell function [64]. Komaki et al. [62] suggested that exosomes play a role in a proangiogenic activity, which is a novel therapeutic approach for treating ischemic diseases. Wang et al. [63] proposed that fetal dermal MSC-exosomes may promote wound healing by activating the adult dermal fibroblast cell motility and secretion ability via the Notch signaling pathway. These may shed light on new aspects of therapeutic strategies based on fetal dermal MSC-exosomes for treating skin wounds. Fan et al. [64] reported that fetal liver MSC-exosomes inhibit the proliferation, activation, and cytotoxicity of NK cells and regulate NK cell function via exosome-associated TGF-β [64].

### 3.3. Clinical Trials Involving hfMSCs

**Figure 1:**
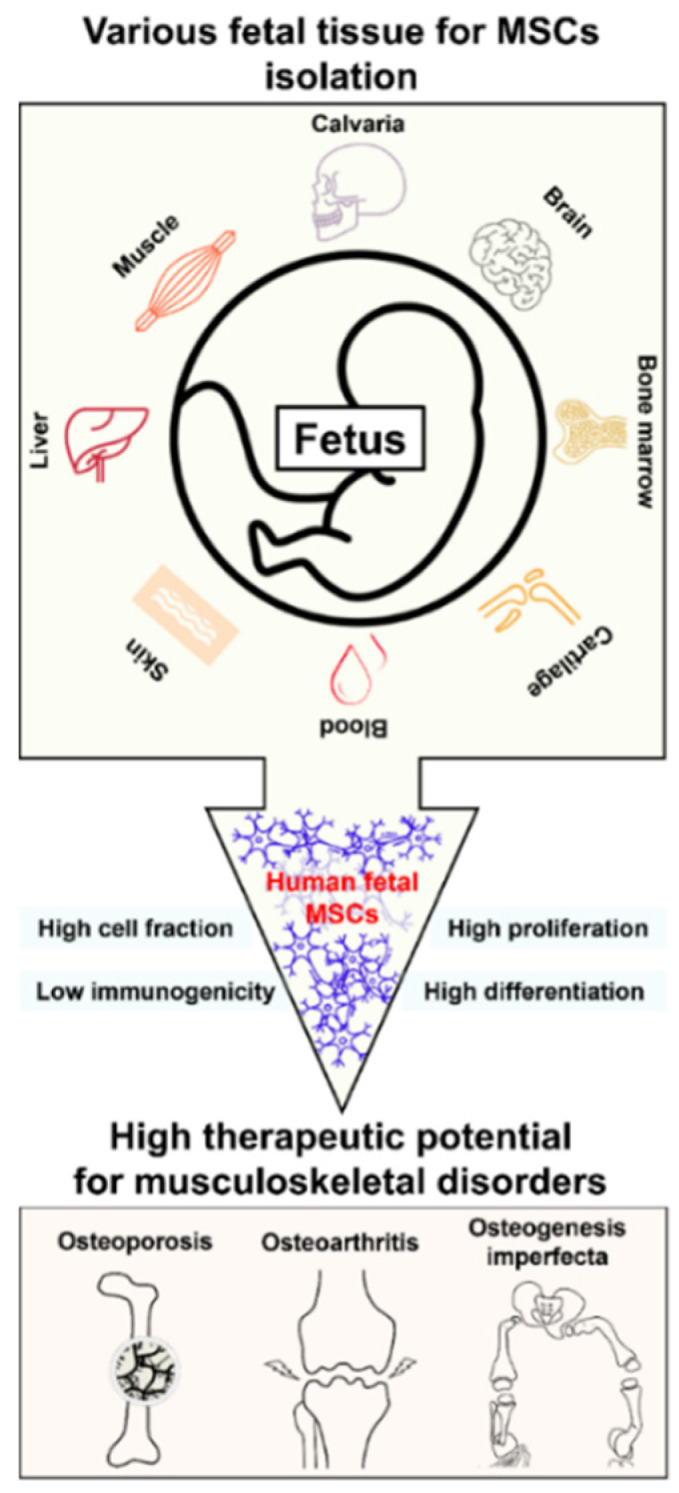
Advantages of human fetal MSCs for the regenerative therapy of musculoskeletal disorders.

Although several clinical trials on hfMSCs are underway, very few have been published. To date, only a few clinical trials involving hfMSCs can be found at clinicaltrials.gov, and only limited information is available on the cell sources and trial conditions to understand the effects and results. Two clinical trials involving fetal MSCs in prenatal cellular therapy for osteogenesis imperfecta have been reported to target type III and type IV OI. These trials involved transplantation of allogeneic MSCs derived from the human fetal liver in the first-trimester (Table 3). They obtained promising outcomes, no new fractures, and improved growth velocity (Figure 1) [50,51].

Applegate et al. reported about bioprocessing of human fetal cells for tissue engineering of skin. They used fetal skin cells with collagen scaffolds, showing a distinction of the fetal skin cells compared to the conventional MSCs in the technical requirements such as collection, culture expansion and storage, and therapeutic feasibility, including skin formation and immunologic reaction. Their cells were made from a master and working cell bank, which confirmed the consistency and safety of cells in the preparation of whole-cell tissue-engineering products [65].

## 4. Future Prospects and Conclusions

The current review highlights the potential application of hfMSCs in the handling of musculoskeletal disorders. The currently reported studies provide evidence for source-dependent differences and similarities based on the hfMSCs origin. Even though hfMSCs have better characteristics than adult MSCs, there are only a few clinical trials for musculoskeletal disorders, including rare skeletal diseases.

### 4.1. Future Prospects of hfMSCs

Even though hfMSCs are relatively new in the field of MSCs, they have gained prominence due to their therapeutic potential and their enhanced multipotency, proliferation, and differentiation capacity compared to adult MSCs. Diverse sources of hfMSCs are available, so it is possible to obtain appropriate and suitable cells for treating target diseases and defects without any side effects. Although adult MSCs have several disadvantages compared to hfMSCs, they have proven to be important therapeutic tools. Similarly, we hope that hfMSCs-based treatments prove to be successful in the field of customized and regenerative medicine and to help in the treatment of rare diseases. An essential factor in cell therapy is immune evasion, which can regulate cellular safety and efficacy to evade the host’s immune response. Thus, it is crucial to further investigate the biological properties of hfMSCs, such as paracrine effects, homing to target locations of injuries and diseases, and immunomodulation, in order to better apply these MSCs as therapeutics. We believe that the disadvantage of hfMSCs compared to other MSCs is the ethical issue, such as protecting human subjects in clinical trials and proper control of stem cells sources for the research. To overcome such adversity, detailed regulations or guidelines should be established in the near future, like those of embryonic stem cells. Once all of these aspects of hfMSCs come to light, we will be in a better position to employ hfMSCs as therapeutic tools in clinical settings.

### 4.2. Conclusions

Ethical issues in hfMSCs research and treatment need to be discussed. However, the regulations and guidelines from those discussions must also ensure the scientific ventures, along with development of appropriate clinical treatment. hfMSCs have advantages such as higher cell fraction with robust cell proliferation, differentiation capacity, and low immunogenicity over other types of MSCs. We believe that the hfMSCs have therapeutic potential for musculoskeletal disorders, including osteoporosis, osteoarthritis, and osteogenesis imperfecta. 

## Figures and Tables

**Table 1:** Information and characteristics of human fetal mesenchymal stem cells (hfMSCs).

Tissue Origin (Age of Donor)	Biological Properties	Phenotype (Positive)	Phenotype (Negative)	Proliferation	Ref.
Blood (Fetus in 16–26 weeks)		CD29, CD44, CD106, CD105, CD73, CD49b, vimentin, laminin, fibronectin	CD45, CD14, CD68, CD34, CD31, HLA-DR, type I collagen	Faster doubling time as every 24–30 h compared to adult No change until 20 passages	[13]
Bone marrow	Adipogenic, osteogenic (Bone marrow derived MSCs > Liver derived MSCs)	CD29, CD44, CD106, CD105, CD73, CD49b, vimentin, laminin, fibronectin	CD45, CD14, CD68, CD34, CD31, HLA-DR, type I collagen		[13,18]
Liver		CD29, CD44, CD54, CD106, CD105, CD73, CD49b, vimentin, laminin, fibronectin	CD45, CD14, CD68, CD34, CD31, HLA-DR, type I collagen		[18]
Lung		CD58, CD71, CD29, CD44, CD54, CD13, CD90, CD105, CD73, CD49e	CD45, CD14, CD31, CD50, CD106, CD11a, HLA-DR,	Stable until 40 passages	[18,19]
Pancreas (Pregnancy in second trimester)	Osteogenic, adipogenic	CD29, CD44, CD13, CD90, CD147, vimentin, type I collagen	CD45, CD34, HLA-DR	Stable until 30 passages	[20,21]
Dermis	Bone, fat, nerve	CD90	CD45, CD34, CD38, CD117, HLA-DR		[22]
Thymus (Pregnancy in second trimester)	Myoblast, chondrogenic osteogenic, adipogenic	CD71, CD44, CD54, CD105, CD90, CD49b, vimentin	CD45, CD34, CD38, HLA Class I, HLA-DR		[23]
Amniotic fluid (Pregnancy in second trimester)	Neural pathway, cardiomyocytes, osteogenic, adipogenic	CD29, CD44, CD105, CD73, CD90, OCT-4, vimentin, type I collagen	CD45, CD14, CD34, CD31, CD106, CD11a, CD13, CD117, HLA-DR		[8,24,25,26,28]
Calvaria	Osteogenic, chondrogenic, adipogenic	CD105, CD90, CD44, CD29	SSEA-3, TRA-1-81, CD34, CD45		Author’s unpublished data

**Table 2:** Advantages of human fetal MSCs (hfMSCs) over adult MSCs.

Biological Property	hfMSCs	Adult MSCs	Ref.
Immune response	HLA-DR * expression after 7 d exposure to IFN-γ	HLA-DR expression after 1 d exposure to IFN-γ	[11,12]
Immunogenic	Less	more	[32]
Proliferation (Population doubling time)	24–30 h	48–72 h	[13,14]
Telomere length	Longer	Shorter	[10]
Osteogenic Differentiation	Higher	Lower	[15,16]
Stemness (Colony-forming)	hfMSCs two times higher than adult MSCs		[15]

* human leukocyte antigen DR isotype.

**Table 3:** Treatment information of four cases of osteogenesis imperfecta (OI).

Year	Patient	Mutation	Source	OI Phenotype	Cell Number (×10^6^)		Outcome	Ref.
					Prenatal	Postnatal		
2005	A	COL1A2, Gly33743Asp	Fetal liver (10 weeks)	III/IV	6.5 hfMSCs at 32 weeks			[50]
2014	B	COL1A2, Gly33743Asp; Gly913Asp	Fetal liver (7 weeks 3 days and 10 weeks)	III	6.5 hfMSCs at 31 weeks (5/kg)	42 at 8 years and 2 months (2.8/kg)	No new fractures, improved growth velocity	[51]
	C	COL1A2, Gly33743Asp; Gly130Asp		IV	4 hfMSCs at 31 weeks (30/kg)	88 at 19 months and 11 days (10/kg)	No new fractures, improved growth velocity	
	D	COL1A2, Gly33743Asp; Gly915Asp		II/III	None	None	Deceased at 5 months of age	

## Data Availability

Not applicable.
